# Does Genetic Predisposition Contribute to the Exacerbation of COVID-19 Symptoms in Individuals with Comorbidities and Explain the Huge Mortality Disparity between the East and the West?

**DOI:** 10.3390/ijms22095000

**Published:** 2021-05-08

**Authors:** Naoki Yamamoto, Rain Yamamoto, Yasuo Ariumi, Masashi Mizokami, Kunitada Shimotohno, Hiroshi Yoshikura

**Affiliations:** 1 Genome Medical Sciences Project, National Center for Global Health and Medicine, Ichikawa 272-8516, Japan; mmizokami@hospk.ncgm.go.jp (M.M.); lbshimotohno@hospk.ncgm.go.jp (K.S.); 2 Department of Drug Development and Regulatory Science, Faculty of Pharmacy, Keio University, Tokyo 105-8512, Japan; ray133@mail.harvard.edu; 3 Division of Retroelement, Joint Research Center for Human Retrovirus Infection, Kumamoto University, Kumamoto 860-0811, Japan; ariumi@kumamoto-u.ac.jp; 4 National Institute of Infectious Diseases, Tokyo 162-8640, Japan; yoshikura@nih.go.jp

**Keywords:** COVID-19, SARS-CoV-2, aggravation, comorbidities, *ACE1* DD genotype, AAT deficiency, ACE2, inflammation, RAAS, Ang II, ADAM17

## Abstract

The elderly and patients with several comorbidities experience more severe cases of coronavirus disease 2019 (COVID-19) than healthy patients without underlying medical conditions. However, it is unclear why these people are prone to developing alveolar pneumonia, rapid exacerbations, and death. Therefore, we hypothesized that people with comorbidities may have a genetic predisposition that makes them more vulnerable to various factors; for example, they are likely to become more severely ill when infected with severe acute respiratory syndrome coronavirus 2 (SARS-CoV-2). To test this hypothesis, we searched the literature extensively. Polymorphisms of genes, such as those that encode angiotensin-converting enzyme 1 (*ACE1*), have been associated with numerous comorbidities, such as cardiovascular disease, hypertension, diabetes, chronic kidney disease, and obesity, and there are potential mechanisms to explain these associations (e.g., DD-type carriers have greater ACE1 activity, and patients with a genetic alpha-1 anti-trypsin (AAT) deficiency lack control over inflammatory mediators). Since comorbidities are associated with chronic inflammation and are closely related to the renin–angiotensin–aldosterone system (RAAS), these individuals may already have a mild ACE1/ACE2 imbalance before viral infection, which increases their risk for developing severe cases of COVID-19. However, there is still much debate about the association between *ACE1* D/I polymorphism and comorbidities. The best explanation for this discrepancy could be that the D allele and DD subtypes are associated with comorbidities, but the DD genotype alone does not have an exceptionally large effect. This is also expected since the *ACE1* D/I polymorphism is only an intron marker. We also discuss how polymorphisms of *AAT* and other genes are involved in comorbidities and the severity of SARS-CoV-2 infection. Presumably, a combination of multiple genes and non-genetic factors is involved in the establishment of comorbidities and aggravation of COVID-19.

## 1. Introduction

Severe acute respiratory syndrome coronavirus 2 (SARS-CoV-2), which originated in China, has become a pandemic, and as of the end of February 2021, the number of infected people worldwide has exceeded 113 million and the death toll is well over 2.5 million (data derived from the WHO Coronavirus Disease (COVID-19) Dashboard, 49TU https://covid19.who.int/tableU49T) (accessed on 25 February 2021). The widespread effects of SARS-CoV-2 infection across the biological systems and organs of patients with coronavirus disease 2019 (COVID-19) are noteworthy. Severe COVID-19 is characterized by an excessive inflammatory response, such as a cytokine storm. Although many infected people, especially young people, remain asymptomatic or have mild symptoms, approximately 19% suffer from more serious effects, including severe pneumonia, which is more common in the elderly and men with pre-existing comorbidities and/or underlying illnesses [1]. This is an interstitial type of pneumonia characterized by “ground glass-like shadows” on plain X-rays and chest CT and can cause serious symptoms, such as acute respiratory distress syndrome (ARDS) and even death [2]. COVID-19 has also been associated with complications such as vasculitis, thrombosis, cerebral infarction, myocardial damage, and multiple organ failure, including acute renal failure [3]. An overt disseminated intravascular coagulation was detected in more than 70% of patients who died from COVID-19 [4].

**Figure 1:**
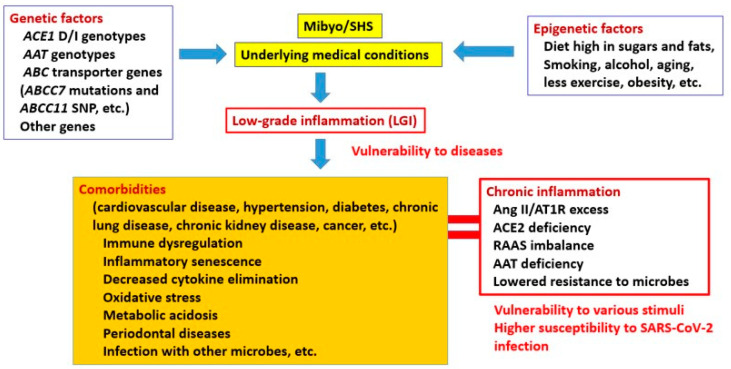
Comorbidities possibly established by interplay among variable genetic and non-genetic factors. Various underlying medical conditions and comorbidities, aging, obesity, and smoking have been reported as risk factors for contracting the new coronavirus infection. Most illnesses are thought to begin with a condition called Mibyo (pre-illness) or Suboptimal Health Status (SHS) [14,15,16], and as it progresses, some physical conditions occur. Comorbidities seem to be established by a complex intertwining of many genetic and environmental factors. Regarding genetic factors, the relationship between comorbidities and *ACE1* genotypes, especially the DD type and AAT deficiency, has been studied extensively (see text). A common feature of patients with underlying medical conditions and comorbidities is mild inflammation (low-grade inflammation, LGI) presenting with dysregulation of the immune system. In the *ACE1* DD genotype, renin–angiotensin–aldosterone system (RAAS) activation and oxidative stress, which lead to immune activation, are considered to be important. The effect of individual gene mutations is mild, but these gene mutations might have a great effect when combined with surrounding genes, or variants of completely different genes, and other non-genetic factors, such as lifestyle-related factors (e.g., a diet high in sugars and fats). Comorbidities are thought to make patients vulnerable to a variety of stimuli because of the many intrinsic problems they have [17,18,19,20]. Especially in the case of SARS-CoV-2 infection, it may be due to their increased susceptibility to such infection.

**Figure 2:**
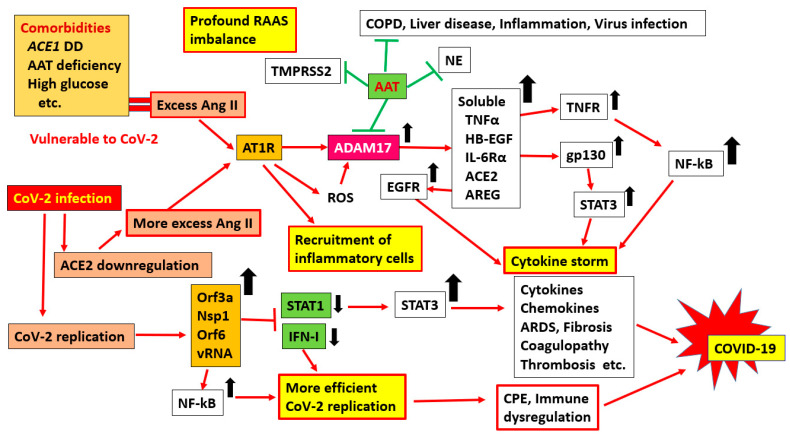
Possible steps leading to severe cases of COVID-19 among individuals with comorbidities after SARS-CoV-2 infection. Upon binding to receptors on target cells, such as epithelial cells and endothelial cells, SARS-CoV-2 induces ACE2 downregulation (endocytosis) along with the endocytosis of viral particles. As a result, Ang II is not subjected to ACE2-mediated degradation, and the ACE1/AT1R axis becomes more dominant. Additionally, it is well recognized that people with the *ACE* DD type show higher ACE1 activity in serum [21]. Thus, when ACE2 deficiency due to SARS-CoV-2 infection occurs, excessive Ang II stimulation will further amplify the RAAS imbalance. Stimulation by Ang II induces the activation of PKC and causes the production of reactive oxygen species and the activation of ADAM17, a pleiotropic enzyme. When ADAM17 is activated, many membrane proteins, such as TNF-α, HB-EGF, IL-6Rα, ACE2, and amphiregulin (AREG), are cleaved. As a result, the activation of proinflammatory substances (IL-6, STAT3, EGFR, TNF-α, and neutrophil elastase) leads to a cytokine storm [22,23,24]. These data support the hypothesis that the RAAS is a major mediator of inflammation. On the other hand, several SARS-CoV proteins have been shown to inhibit multiple arms of type I IFN responses and significantly suppress IFN-β production during infection. Utilizing these strategies, SARS-CoV-2 may replicate more efficiently in infected individuals. AAT suppresses SARS-CoV-2 infection through the inhibition of TMPRSS2 and ADAM17 protease. AAT-mediated ADAM17 inhibition can also regulate ACE2 cleavage and protect lung and heart tissue from the harmful effects of RAAS imbalance. In addition, AAT inhibits ADAM17, resulting in the inhibition of TNF-α and IL6-R cleavage, which can exert anti-inflammatory effects. In addition, AAT is involved in the control of neutrophil chemotaxis and the suppression of neutrophil elastase (NE). An upward and a downwards black arrows indicate an increase and a decrease in protein, respectively.

The human *ACE1* gene, located on chromosome 17, has a polymorphism consisting of an insertion (I) or deletion (D) of a 287-base-pair (bp) Alu repeat sequence in intron 16 [5], known as the I/D polymorphism, and there are three different genotypes: II, ID, and DD. Of particular importance is the fact that ACE2 is a receptor for SARS-CoV-2 [6]. We recently reported significantly different infection and mortality rates for SARS-CoV-2 between European and East Asian populations, and this correlated with the *ACE1* D/I genotype [7,8]. We showed that the European population has an *ACE1* II genotype frequency that is approximately twice as low as that of the Asian population and has a higher prevalence of and mortality due to COVID-19. Similarly, Pati et al. reported an *ACE* deletion allele associated with COVID-19 prevalence and mortality in Asians [9]. Hatami et al. also showed, in their meta-regression analysis, that *ACE1* I/D polymorphism may affect COVID-19 recovery rate worldwide [10]. In addition, AAT-deficient alleles may contribute to national and global differences in COVID-19 infections and their associated severity and mortality [11,12,13]. As of the end of February, 2021, the numbers of deaths per million in Europe and the Americas were about 43 and 21 times higher than that in East Asia, respectively (data derived from the WHO Coronavirus Disease (COVID-19) Dashboard, 49T https://covid19.who.int/table49T) (accessed on 25 February 2021). By contrast, it has become apparent that there is little difference among children and adults without underlying illnesses in these regions, as they are less likely to have severe symptoms, regardless of their location. Thus, this huge mortality disparity between the East and the West seems to be the key to explaining the exacerbations of SARS-CoV-2 infections. Therefore, we hypothesized that some people have genetic factors that predispose them to comorbidities, namely, chronic inflammatory conditions, and make these individuals more vulnerable to SARS-CoV-2 infection and its aggravation (Figure 1 and Figure 2). Hence, we focused on mutations in host genes associated with comorbidities and inflammation, especially *ACE1* and *AAT*. 

## 2. Pre-Existing Conditions and Inflammation

A common feature of older people and those with pre-existing medical conditions, such as cardiovascular disease, diabetes, chronic respiratory disease, and cancer is low-grade inflammation (LGI) [25], which is a symptom exhibited by all high-risk people who develop severe COVID-19 (Figure 1). Although inflammation is a long- and well-established symptom of many infectious diseases, molecular and epidemiological studies have increasingly demonstrated the roles played by inflammation in a wide range of noninfectious diseases [26,27]. LGI is characterized by a chronic increase in inflammatory cytokines, such as interleukin-6 (IL-6), tumor necrosis factor alpha (TNF-α), and interleukin-1 beta (IL-1-β). Although inflammatory cytokines function as a biological defense mechanism, they are a double-edged sword. Multiple genetic and/or environmental factors appear to be involved in the development of most disorders, including allergies and autoimmune diseases, and are one of the most important challenges facing modern medicine. 

In a proper immune response, immune cells are recruited to the injured area via proinflammatory signaling pathways [28]. These acute inflammatory events typically resolve relatively quickly, and the level of inflammation returns to baseline. However, if the inflammation is not resolved, it becomes chronic and persists. Chronic inflammation is a condition of excessive proinflammatory signaling in which the elimination phase is not achieved (Figure 1). Under chronic inflammation in the body’s organs, such as the lungs, the phase during which parenchymal cells regenerate does not occur at the appropriate time, and instead, these cells are replaced by proliferated fibroblasts and collagen fibers [29]. The arrangement of the vascular system is also disrupted, and the resulting circulatory disruption is likely to cause more parenchymal cell damage. Thereby, the function of the entire organ is reduced, and it becomes hard and atrophied, due to fibrosis. This condition is called pulmonary fibrosis, cirrhosis, or nephrosclerosis, depending on the organ involved, and can lead to organ failure.

Metabolic and lifestyle factors are strongly associated with the development of comorbidities, such as obesity, hypertension, type 2 diabetes, and increased peripheral inflammation [29]; thus, much research has been conducted to investigate these relationships. Obesity alters the metabolic and endocrine functions of adipose tissue and increases the release of the fatty acids, hormones, and proinflammatory molecules that contribute to obesity-related complications [30]. During acute colitis, TNF-α, IL-1β, and IL-6 mRNA levels are significantly increased in mesenteric adipose tissue, and the downregulation of TNF-α maintains insulin sensitivity [31]. The excessive release of proinflammatory mediators, such as TNF-α and IL-6, may be due to the accumulation of macrophages in the adipose tissue of obese individuals [32]. Indeed, macrophage numbers increase in obesity, and macrophages participate in inflammatory pathways that are activated in the adipose tissue of obese individuals. The release of proinflammatory cytokines is also associated with the consumption of a high-fat diet, which increases the production of IL-1, IL-6, and TNF-α by increasing nuclear factor kappa B (NF-κB) levels [33]. In addition, hyperglycemia seems to acutely exacerbate the innate cell inflammatory status, resulting in endothelial adhesion and potential vascular damage [34]. In obese patients with type 2 diabetes, hypertension is associated with increases in insulin resistance and IL-6 levels [35]. In patients with type 2 diabetes, NLRP3 inflammasome activation is elevated in myeloid cells [36]. Furthermore, increasing evidence indicates that common risk factors for brain disorders, including atherosclerosis, diabetes, hypertension, obesity, and infection, involve the activation of NLRP3, NLRP1, NLRC4, and AIM2 inflammasomes, which are also associated with various neurological diseases [37].

Aging is the most common risk factor for developing COVID-19 [38]. As individuals age, gradual deterioration, known as immune senescence, occurs in the peripheral and central components of the immune system, increasing susceptibility to infections and illnesses. Changes in the expression of several immune-related genes have been observed in different regions and tissues of the elderly, and this pattern is exacerbated by SARS-CoV-2 infection [39]. In addition, since the fatality rate of COVID-19 is higher in men than in women, gender differences have also been the subject of research. The gene encoding ACE2, the receptor for SARS-CoV-2, has protective effects against COVID-19 and is located on the X chromosome. As women have two copies of the X chromosome, the favorable effects of ACE2 alleles are increased in women. Due to men having only one X chromosome, most X-linked syndromes are male diseases, and extensive research has been focused on sex-related aspects, such as the effects of female sex hormones [40]. In addition, immune-stimulatory genes, including those encoding Toll-like receptors (TLRs), ILs, and microRNAs, present on the X-chromosome may contribute to the lower rates of infectivity and mortality of SARS-CoV-2 in women [41].

## 3. Association between Genetic Predispositions and Comorbidities 

In COVID-19, underlying diseases and physical and mental disorders, such as cardiovascular disease, hypertension, diabetes, chronic lung disease, chronic kidney disease, cancer, and obesity, as well as factors such as aging and smoking, have been reported as risk factors for aggravation. Both genetic and environmental factors may be involved in the etiology of many of these comorbidities (Figure 1) [21,42,43,44,45]. The genetic predispositions described below may be related to comorbidities. Interestingly, polymorphisms in these genes show frequencies that significantly differ between Westerners and East Asians.

### 3.1. ACE1 D/I Genotype

An association between the renin–angiotensin–aldosterone system (RAAS) and phenotypic expression of various comorbidities has been reported [46,47,48]. The RAAS exerts its effects with the involvement of the liver, lungs, adrenal glands, kidneys, and vascular smooth muscle to maintain normal blood pressure and Na levels in the body [49,50]. The secretion of renin from the renal juxtaglomerular apparatus is triggered by decreased renal perfusion pressure, sympathetic nerve excitement, and decreased blood Na levels. Renin is a protease that acts on angiotensinogen in the blood to produce angiotensin I (Ang I). Ang I is then converted to the octapeptide angiotensin II (Ang II) by ACE1 on the vascular endothelial cell membrane. In the classical RAAS, the ACE1–AngII–angiotensin II receptor type 1 or the AT1 receptor (AT1R) axis promotes vasoconstriction to maintain blood pressure, fluid intake, and Na retention, in collaboration with aldosterone secretion. In some pathological conditions, the excessive activation of this axis can have harmful effects, such as increased oxidative stress, fibrosis, cellular growth, and inflammation. By contrast, ACE2, an ACE1 homolog, is involved in the production of ANG (1–7) and sends negative stimuli to cells via MasR. This is an oversimplified model, as the RAAS is considerably more complicated. 

RAAS activation, along with oxidative stress, inflammation, and the activation of particular immune cells, may contribute to the close relationship between hypertension and diabetes [51]. In diabetes, vascular inflammation, atherosclerosis, endothelial dysfunction, vascular fibrosis, and arterial remodeling are thought to cause micro- and macrovascular diseases, leading to the onset of cardiovascular diseases [51]. Furthermore, the relationship between the sympathetic nervous system and stress and the interaction between Ang II and the sympathetic nervous system are well-known classical mechanisms that are involved in the maintenance of homeostasis. The DD genotype in the *ACE1* gene has been shown to be associated with an increased risk of developing numerous common adult diseases, including hypertension, diabetic nephropathy, severe hypoglycemia in diabetes, cardiovascular diseases, myocardial infarction, and ischemic heart disease as well as symptoms of various other cardiac endpoints, pre-eclampsia, cerebral infarct, encephalopathy, asthma, cancer, and psychiatric diseases [52,53]. Both genetic and lifestyle factors may contribute to the onset of cardiovascular disease, which is a major cause of morbidity and mortality in the West [54]. In addition, the upregulation of *ACE1* gene expression has been observed in the myocardia of patients with heart failure [55]. The *ACE1* I/D polymorphism is also associated with pulmonary arterial pressure in Caucasian patients with chronic obstructive pulmonary diseases (COPDs), including chronic bronchitis and emphysema [56]. In addition, the high ACE1 activity of carriers of the DD genotype may play an important role in increasing plasma PAI-1 levels, suggesting that genetic mutations in *ACE1* may alter the balance in fibrinolytic pathways [57]. In addition to the cardiovascular system, genotypes containing the D-allele, especially DD, have been reported to be associated with several different underlying medical conditions and comorbidities that have been reported as risk factors for the aggravation of COVID-19, as well as aging, obesity, and smoking [58,59].

### 3.2. AAT

In SARS-CoV-2 infection, AAT suppresses the key protease Transmembrane Serine Protease 2 (TMPRSS2), which cleaves the viral spike (S) protein to facilitate infection, and thus inhibits viral infection [60]. AAT also inhibits the ADAM metallopeptidase domain 17 (ADAM17) [12]. AAT deficiency is a hereditary disorder associated with early-onset COPD and liver disease [61,62]. AAT deficiency is not a rare disease among Caucasian individuals in Northern Europe and immigrants from these countries to the New World. According to de Serres and Blanco, in a total population of 4.4 billion in 58 surveyed countries, there are at least 116 million carriers (those with the Pi phenotypes PiMS and PiMZ) and 3.4 million with deficiency allele combinations (the phenotypes PiSS, PiSZ, and PiZZ) for the two most prevalent deficiency alleles PiS and PiZ. More than one-third of adults with “PI * ZZ” type AAT deficiency have significant underlying liver fibrosis [63]. The highest prevalence is 114 per thousand members of the population for PI*S in Portugal and 104 in Spain [64]. Over the past decade, it has been demonstrated that AAT is a broad-spectrum anti-inflammatory, immunomodulatory, anti-infective, and tissue-repair molecule [62]. Therefore, although AAT deficiency is not a rare disease, it is rarely diagnosed [65]. New data suggest that AAT deficiency may be one of the most common and serious single-locus genetic disorders worldwide [64].

AAT is produced in the serum as an acute phase reacting substance together with C-reactive protein (CRP), mannose-binding protein, fibrinogen, and haptoglobin as a result of the action of inflammatory cytokines in the liver [66]. Regarding the pathophysiology of COPD, it is thought that neutrophils and their products—elastase, cathepsin G, and proteinase-3—attack the airways, leading to inflammatory changes [67]. Patients with a genetic AAT deficiency lack control over inflammatory mediators, such as IL-1β, IL-6, TNF-α, and IL-8, which increases the incidence of various types of vasculitis [68]. These mediators induce many pathological features associated with the disease, such as emphysema, increased neutrophils in the airways, and the hypersecretion of mucus [69]. In addition, studies of AAT-deficient adults with the Pi * ZZ mutation and Pi * Z-overexpressing mice have found fatty livers and impaired lipid secretion. In this genetic liver disease, misfolded mutant AAT protein may not be secreted but is, instead, accumulated in hepatocytes, causing toxicity [70]. Of particular importance is the unique sensitivity of AAT-deficient individuals to certain chemicals and particulate environmental agents [65]. Such exposure can cause diseases of the liver and lungs, as well as other adverse health outcomes.

AAT is a constitutive tissue protectant and an antiviral and anti-inflammatory molecule. As described previously, AAT suppresses the key protease TMPRSS2 and inhibits viral infection [60]. AAT also inhibits ADAM metallopeptidase domain 17 (ADAM17) [12], as discussed below in more detail. In addition to its antiprotease activity, since AAT has anti-inflammatory and immunomodulatory functions, its use as a therapeutic has been considered for other inflammatory conditions, such as rheumatoid arthritis, diabetes, cystic fibrosis, and asthma [71].

### 3.3. Other Genes and Their Subtypes

#### 3.3.1. FXIIIB and PV92

Other interesting candidates are the D/I polymorphisms of the *FXIIIB* and *PV92* genes, which, as in the *ACE1* gene, occur in Alu insertions and behave similarly to *ACE1* D/I [72]. FXIIIB is a coagulation factor in plasma, and PV92 (CDH13) is associated with cadherin and hypertension [72,73]. Therefore, there seems to be a strong connection between these genotypes and the symptoms associated with COVID-19. A comprehensive analysis using cells derived from 42 Japanese and European individuals (including 2 Africans) conducted as a preliminary study showed that the D/I types of these three genes (*ACE1*, *FXIIIB*, and *PV92*) were overwhelmingly II or ID in Japanese individuals, whereas there were many DD or ID types present among Europeans (Ariumi et al., unpublished). This indicates that the *ACE1* D/I gene polymorphism and the *FXIIIB* and *PV92* gene D/I polymorphisms are likely to overlap. 

#### 3.3.2. Neanderthal Haplotype

Recently, Zeberg and Pääbo showed interesting data indicating that the risk of developing severe cases of COVID-19 is increased by a genomic segment around 50 kilobases in size (the genetic variants on chromosome 3) that is inherited from Neanderthals and is carried by around 50% of people in South Asia and around 16% of people in Europe [74]. Very interestingly, this trait seems to be rarely inherited by East Asians, where the damage caused by the new coronavirus has been much lower than its effects in Europe and the Americas [61]. We already discussed our hypothesis that East Asians may have genes that confer resistance to SARS-CoV-2 infection and that people in East Asia may have been hit by multiple outbreaks of infectious viruses similar to coronaviruses in the past [8]. In fact, East Asia has experienced SARS and Middle East respiratory syndrome (MERS) in this century alone. Similarly, Zeberg and Pääbo suggested the possibility that the haplotype has decreased in frequency in East Asia owing to negative selection, perhaps because of the presence of coronaviruses or other pathogens. Future analysis is expected to investigate how the haplotype of the Neanderthal DNA is involved in the exacerbation of COVID-19 symptoms, including its relationship with host immunity.

#### 3.3.3. Human Leukocyte Antigen (HLA) 

Human leukocyte antigen (HLA) is a protein of the immune system that is involved in antigen presentation. It has received particular attention in relation to disease susceptibility, and it is well known that the composition of HLA types varies greatly among countries and ethnic groups. *HLA* polymorphisms are associated with susceptibility to various diseases, such as autoimmune diseases and infectious diseases. Although studies have been conducted in Italy and Spain, to date, there is no evidence that a particular HLA type is associated with the exacerbation of COVID-19 symptoms [75]. Further research is necessary to explore this possible association.

#### 3.3.4. ATP-Binding Cassette (ABC) Transporter Genes

The first described eukaryotic ATP-binding cassette (ABC) protein, which was isolated in 1986, was MDR1 [76], a multidrug transporter responsible for multidrug resistance in cancer cells. There are 48 *ABC* transporter genes in the human genome, and abnormalities in these genes cause various diseases, indicating the important roles they play in maintaining human health [77]. One of these ABC transporter genes, *ABCC11*, is responsible for determining whether earwax is dry or wet. The proportion of people with wet earwax is overwhelmingly high among Westerners and Africans but is very low in East Asians [78]. It is believed that ancient northeastern Eurasians acquired a mutation (538G→A) in this gene during migration that increased the frequency of dry earwax. Since then, this population has expanded, peaking in North China and South Korea, and is thought to show the current geographical extent among East Asians [78]. Interestingly, those with the wild type of *ABCC11* (538G) have osmidrosis, whereas those with the mutated *ABCC11* (538A) do not. Although axillary osmidrosis is not related to health, its unique physical characteristics are related to the active excretion of fat and other substances from the body; therefore, it may be related to some health conditions, and it may be interesting to analyze the association between SARS-CoV-2 infection and this genetic polymorphism.

#### 3.3.5. Epidermal Growth Factor Receptor (EGFR)

Finally, we would like to add that the characteristics of EGFR may also explain why SARS-CoV-2 kills so few people in East Asia. Klann et al. reported that the inhibition of EGFR signaling prevents SARS-CoV-2′s replication [79]. Major interethnic differences in the allelic frequencies of the *EGFR* intron 1 polymorphism exist. 

In sum, the data obtained to date do not rule out the involvement of mutations in genes other than *ACE1* and *AAT* in the exacerbation of COVID-19 symptoms. Rather than focusing on individual genes, a comprehensive consideration of these genes will help us to better understand the risk factors related to COVID-19 in the future.

## 4. The Effect of the ACE1 DD Genotype Alone Seems to Be Modest

As previously stated, there are numerous reports that genotypes containing the D allele, especially DD of *ACE1*, are associated with a variety of comorbidities. However, there are also quite a few reports of large prospective studies showing that this *ACE1* gene polymorphism does not pose a perceptible risk [80,81]. In some meta-analyses, the involvement of the DD genotype in hypertension, left ventricular hypertrophy, cardiomyopathy, and restenosis after percutaneous transluminal coronary angioplasty is controversial. Furthermore, there are reports that D alleles and DD homozygotes may be important genetic molecular markers of COPD susceptibility in Asians but not in Europeans [82,83]. For well-studied cardiovascular diseases, much of the evidence available supports the idea that the DD *ACE1* genotype adversely affects certain cardiovascular diseases but only in certain geographic areas. Various factors, particularly those that are specific to certain subgroups of patients, appear to give rise to these discrepancies. It is not yet known whether these discrepancies are due to the interaction between the *ACE1* DD genotype and other genes or a biochemical mechanism that has not yet been elucidated. However, it is important to note that the *ACE1* DD genotype is unlikely to have a significant effect as a single factor. ACE1 is a component of the RAAS cascade, and the DD polymorphism is an intron marker. Moreover, we should keep in mind that approximately 55% of individuals carry the D allele [84]. This seems to indicate that one such genetic mutation does not have more than a slight effect on complex multifactorial diseases such as cardiovascular disease, diabetes, and even renal disease. 

It has been established that high blood pressure, elevated serum cholesterol, obesity, smoking, male sex, atherosclerosis, and diabetes are associated with an increased risk of fatal outcomes, such as cardiovascular events, including myocardial infarction, stroke, and kidney failure. Heart attacks are the largest single cause of death in Western industrialized nations, where many DD carriers exist [54]. By contrast, there have been reports that many centenarians are DD carriers [85]. What this means is that the effect of the DD genotype or D allele alone is likely weak or modest. This, again, seems to suggest that the DD genotype and D-alleles may have significant effects only when combined with other genes near to the *ACE1* gene or other variants of a completely different gene. We think the notion that the effect of the D-allele is not negligible but weak and not immediately noticeable is particularly important. The overall picture is more complicated than what can be explained by the D-allele alone. However, the possession of the D-allele may be a precondition that makes one more vulnerable to major events affecting health (Figure 1 and Figure 2). This means that the D-allele, whose effect is small on its own, quietly but firmly plays an important role in the development of comorbidities, and it can result in severe outcomes when combined with another factor (in this case, SARS-CoV-2 infection). 

The same idea could be applied to other gene mutations, such as that causing AAT deficiency. When countries were grouped into those with high prevalence of AAT deficiency and those with low prevalence, though the numbers of the patients or the deaths were proportional to the population sizes within each group and the case–fatality rates were ~0.01 for both groups, the number of the patients or the number of deaths per head of population was ~10-fold higher for countries with a high prevalence of AAT deficiency; i.e., the outbreak size was 10-fold larger for countries with a high prevalence of AAT deficiency. Interestingly, the geographical distribution of AAT deficiency almost overlaps that of the Neanderthal trait [74].

## 5. SARS-CoV-2 Infection and an Excessive Inflammatory Response

Based on the detection of high levels of cytokines, a “cytokine storm” has been proposed as the cause of severe COVID-19 cases (Figure 2) [22]. Similar findings have been reported in critically ill patients with severe acute respiratory syndrome (SARS) [23]. Ang II, a key component of the RAAS, is also known to function as a proinflammatory cytokine [86]. Ang II increases vascular permeability, which initiates the inflammatory process via AT1R. Ang II may also contribute to the recruitment of inflammatory cells to tissues through the regulation of adhesion molecules and chemokines by resident cells. Injecting Ang II into mice induced medial vascular hypertrophy in the heart, kidneys, and aorta as well as perivascular fibrosis, cardiac hypertrophy, and hypertension in the heart and kidneys [87]. In addition, the activation of Ang II is associated with vascular remodeling through the transactivation of epidermal growth factor receptor (EGFR), which induces oxidative stress in the ER [88,89]. These data support the hypothesis that the RAAS is a major mediator of inflammation. Furthermore, in a mouse model of acute respiratory distress syndrome (ARDS), the expression of recombinant ACE2 was shown to counteract the downregulation of ACE2, thereby suppressing the development of lung injury [90]. Therefore, it is highly likely that the RAAS is involved in the development of ARDS after SARS-CoV-2 infection. Ang II is also involved in tissue repair and remodeling through the regulation of cell proliferation and matrix synthesis.

It is important to note that the binding of Ang II to AT1R also induces the pleiotropic inflammatory molecule ADAM17 [91]. Ang II stimulation induces an increase in Ca^++^, the activation of protein kinase C (PKC) via inositol trisphosphate (IP3) and diacylglycerol (DAG), vasoconstriction, and aldosterone secretion as well as active oxygen production and ADAM17 activation [92]. Increased levels of Ang II and its binding to AT1R during hypertension are especially important in the activation of ADAM17. The activation of ADAM17 results in increased membrane protein cleavage. Cytokines such as TNF-α, interferon (IFN)-γ, transforming growth factor (TGF)-β, IL-4, IL-10, IL-13, IL-6, and fractalkine are the major substrates of ADAM17, and their regulatory modulation causes inflammation (Figure 2). Other ADAM17 substrates include growth factors (TGF-α and Heparin-binding epidermal growth factor-like growth factor (HB-EGF)), adhesion molecules (L-selectin, ICAM-1, and VCAM-1), receptors (TNFR, EPCR, and IL-6R), and enzymes (ACE2 and neprilysin) [93]. This suggests that the activation of ADAM17 modifies inflammatory symptoms in a wide variety of ways. When sIL-6Rα is produced, the sIL-6R-IL-6 complex transduces an intracellular signal by binding to gp130, which is expressed in nonimmune cells, such as vascular epithelial cells, fibroblasts, and alveolar epithelial cells [22,93]. 

Importantly, SARS-CoV-2 infection induces a downregulation of ACE2 endocytosis along with the endocytosis of viral particles when the viral S protein binds to receptors on target cells, such as epithelial cells and endothelial cells [60]. As described above, Ang II also increases ADAM17 expression in cardiomyocytes and induces a reduction in ACE2 at the cell surface by ACE2 cleavage. As a result, ACE2 expression is reduced on the cell surface, the conversion of Ang II to Ang (1–9) and Ang II to Ang (1–7) becomes inadequate, and hypertension and various other harmful symptoms follow. A reduction in these protective peptides, which is the cornerstone of negative regulation via ACE2–AT2R, further increases the Ang II level. As a result, the imbalance of ACE1/ACE2 is increased, and the subsequent activation of proinflammatory substances, such as IL-6, signal transducer and activator of transcription (STAT), EGFR, TNF-α, and neutrophil elastase (NE), causes a cytokine storm (Figure 2) [22,23]. In this situation, IL-6 activates STAT3 in immune cells, such as macrophages and lymphocytes, via IL-6Rα. Furthermore, sIL-6Rα forms a complex with IL-6 and then acts on various cells expressing gp130, including vascular endothelial cells, to activate STAT3. Activated STAT3 acts on NF-κB to form an amplification loop that further enhances its activation (Figure 2) [22]. When SARS-CoV-2 infection occurs, the imbalance of ACE1/ACE2 is disastrous. 

By contrast, there are data that suggest a role for AAT in preventing the exacerbation of COVID-19 [11,12,13,94]. When the SARS-CoV-2 spike (S) protein binds to the ACE2 receptor, it is coupled with the membrane protein TPMRSS2 and the endosomal cysteine proteases cathepsin B and L (CatB/L) [22]. The S protein is cleaved, resulting in the fusion of the viral and host cell membranes, and the virus invades the host cell [60]. AAT suppresses this effect in a dose-dependent manner. As the infection progresses, the level of AAT, along with the levels of other tissue proteases, increases as part of the immune response. These related antiviral and anti-inflammatory reactions may not be effective when AAT deficiency is present. Importantly, AAT binds to cholesterol-rich lipid rafts and blood lipid particles and directly to IL-8, ADAM17, and damage-associated molecular pattern (DAMP) molecules [66,92]. Since these activities are abolished by smoking, high glucose levels, and bacterial proteases, a relative AAT deficiency can also be acquired [95]. This activation of ADAM17 is also intricately linked to the RAAS, because the treatment of cardiomyocytes with Ang II induces an AT1R-mediated increase in the expression and activity of ADAM17 and a loss of ACE2 [96]. Therefore, serum Ang II levels increase as ACE2 cell surface expression decreases, further enhancing the ACE1/ACE2 imbalance.

AAT is an acute phase protein with antiviral activity and is thought to be elevated in the blood, even with SARS-CoV-2 infection. However, this anti-inflammatory response is overwhelmed in critically ill patients, owing to the significantly high IL-6 levels relative to the levels of AAT. In patients with COVID-19, an increased IL-6/AAT ratio was correlated with poor prognosis, while a reduced IL-6/AAT ratio was associated with clinical resolution [97].

## 6. Viral Load and Disease Severity

We have provided evidence that variants of some host genes are directly or indirectly related to comorbidities and are strongly associated with immune dysregulation, which may result in increased vulnerability of the host to various stimuli. When SARS-CoV-2 infects such vulnerable individuals, possibly as a result of the presence of comorbidities, the virus may be more likely to propagate than in a host without these variants and associated comorbidities, thus making it more pathogenic and inflammatory (Figure 2). In SARS-CoV-2 infection, alveolar epithelial cells, macrophages, and circulating monocytes are activated via pattern recognition receptors (PRRs) that recognize viral products and produce large amounts of inflammatory cytokines and chemokines [24]. Monocytes and T cells are involved in extensive lung inflammation. The postmortem pathology of COVID-19 patients shows severe lymphopenia with the inflammatory infiltration of interstitial mononuclear cells in the lungs and activated T cells [98]. Furthermore, patients with COVID-19 also have lower levels of regulatory T cells, and the lungs are more obviously damaged in severe cases [99].

In addition to self-renewal machinery, SARS-CoV-2 has evolved various means of countering the host innate immune system [89,100]. Some viral proteins antagonize the antiviral activity of IFN and its downstream activation of the Janus kinase (JAK)–STAT signaling pathway. After the virus binds to ACE2, the RNA genome of SARS-CoV-2 enters the cytoplasm of the host cell via endocytosis or direct membrane fusion [60,101,102]. The positive-sense RNA genome is translated by the host translation machinery to make polyproteins that are cotranslationally cleaved by proteases encoded by the polyprotein to generate components of the RNA-dependent RNA polymerase (RdRp) complex. The SARS-CoV-2 genome contains a large 5′ open reading frame (Orf) that encodes 16 nonstructural proteins (Nsps). The structural proteins’ spike, envelope, membrane, and nucleocapsid are encoded at the 3′ end of the genome. Nonstructural accessory proteins (6 Orfs), such as Orf6, are also encoded by these genes [101,102].

In SARS, a high serum viral load and the detection of the virus at multiple sites is predictive of adverse clinical outcomes [103], and in MERS, a high viral load in respiratory samples is associated with more serious illness. Interesting data on SARS-CoV-2 were recently reported by Hagman et al. [104]. They found that the detection of SARS-CoV-2 RNA in the serum at admission was associated with a seven-fold increased risk of critical disease and an eight-fold increased risk of death in a cohort of 167 patients hospitalized for COVID-19. These findings indicate a close link between persistent uncontrolled viral replication and disease aggravation. For COVID-19, the viral load in the oropharynx also appears to increase with age [105]. Consistent with these reports, SARS-CoV-2 RNA was detected in the sera of 5 of 17 critically ill patients, two of whom died, but was not detected in any of the 31 patients with milder cases of COVID-19 [105]. In addition, in a very recent study, SARS-CoV-2 RNA was detected with an ultrasensitive polymerase chain reaction (PCR) method in the sera of 74% (43/58) of COVID-19 patients tested, and the level of SARS-CoV-2 RNAemia was associated with severity [106]. The presence and level of SARS-CoV-2 RNA in serum were correlated with the disease severity, and the risk of poor outcomes tended to be higher in the serum-positive group. From a virological perspective, it makes sense that the viral load parallels the symptom intensity. Therefore, the inability of the host to adequately control viral replication and disseminated hematogenous spread to multiple organs is likely to be the driving force behind the inflammatory response that characterizes COVID-19 aggravation.

IFN is probably the most frequently studied molecule that inhibits viral replication. Several SARS-CoV-2 proteins have been shown to inhibit multiple arms of the type I IFN response and significantly suppress IFN-β production during infection [89]. IFN-β signals in an autocrine and paracrine manner and activates the IFN-stimulated gene (ISG) via JAK/STAT signaling. Nsp1 inhibits the phosphorylation of STAT1, and Orf6 inhibits the nuclear translocation of STAT1, further suppressing the production of the ISG protein. The N protein of SARS-CoV-2 also inhibits the recognition of foreign viral RNA by inhibiting the TRIM25-induced activation of RIG-I and IRF3 phosphorylation [89,101,107,108]. Through these or additional strategies, SARS-CoV-2 infection may occur more efficiently in some infected individuals. Factors that promote virus growth should also be considered in addition to the suppression of IFN. However, since that is outside the scope of this paper, we did not cover the topic extensively. 

## 7. Discussion and Conclusions

The pathomechanisms of comorbidities are closely related to chronic inflammation, and severe COVID-19 is characterized by an excessive inflammatory response, such as a cytokine storm. We and others have shown that some gene mutations, such as the *ACE1* DD genotype and AAT-deficient alleles, may contribute to geographical disparities in COVID-19 severity and mortality [7,8,9,11,12,13]. Patients with several comorbidities experience more severe COVID-19 symptoms than patients without the *ACE1* DD genotype or AAT-deficiency. These data prompted us to hypothesize that people who are prone to becoming more severely ill have often already been suffering from comorbidities prior to SARS-CoV-2 infection, which may make them vulnerable to various burdens. However, several data suggest that the effects of these genetic mutations alone are modest, and their combination with various other genetic and epigenetic factors seems to contribute significantly to the onset of comorbidities. In the present pandemic, people with underlying comorbidities are being more gravely hit by SARS-CoV-2 infection, allowing the virus to trigger more severe cases of COVID-19, but more importantly, patients with comorbidities are just as likely to be vulnerable to burdens other than viruses. If this is the case, future measures for comorbidities will be even more important.

With respect to the involvement of gene mutations in comorbidities, even moderately rare mutants can have strong effects, and many typical monogenic diseases follow Mendel’s laws of inheritance. In some patients infected with SARS-CoV-2, the disease becomes severe, even though there is no underlying medical condition. Thus, it is necessary to consider the possibility of a rare dominant mutation. Furthermore, the concept of susceptibility to COVID-19 becomes more complex when considering potential interactions between genetic and environmental factors, such as smoking and obesity. The host–parasite relationship is the basis of microbiology, and SARS-CoV-2 infection is no exception. Whether a SARS-CoV-2 infection reaches the onset stage of COVID-19 or becomes severe depends on various factors, such as host resistance and the pathogenicity of the virus. On the host side, susceptibility and resistance are the major determinants, while on the viral side, various factors, such as the type and amount of virus, its pathogenicity, and the infection site are the important deciding factors. Recently, mutant strains of SARS-CoV-2 that are more pathogenic have emerged in the United Kingdom and South Africa and have rapidly spread to other regions [109]. This is very important in terms of the current vaccines’ efficacy and future epidemic prediction. However, these mutant strains are beyond the scope of this review; hence, we did not address these issues.

An American epidemiologist, Finestein, first introduced the term comorbidity in the 1970s [110]. Along with lifestyle-related issues and aging, this is one of the most important challenges facing medical research today. In oriental medicine, the condition where “the risk of getting sick is increasing” is called “Mibyo” [14] or Suboptimal Health Status (SHS) [15,16]. Mibyo, or SHS, does not clearly distinguish health and illness as a dichotomy. Instead, the state of the mind and body is perceived as a continuum between health and illness, and it represents all the processes within. In the processes of being born, growing, and aging, humans encounter various physical challenges, but the literal translation of Mibyo, or SHS, is “not yet ill”. Thus, this is a state of “progressing toward illness”, and over time, it will definitely be in a state of being given a “disease name”. This may mean that some physical distress will occur even before the medical condition emerges. Mibyo, or SHS, is the gateway to the development of an individual chronic disease, and when two or more such diseases are simultaneously present in a patient, the state is known as a comorbidity. The significance of comorbidities is that they account for nearly all causes of human death, including cancer. This indicates the importance of responding appropriately to comorbidities, including Mibyo/SHS. Comorbidities are conditions involving mild chronic inflammation, and the relationship between inflammation and the onset of comorbidities is an unavoidable issue, including the Mibyo/SHS, which shows almost no subjective symptoms. Therefore, if our assumption that the comorbid conditions manifested in high-risk individuals and their vulnerability to them are based on genetic predispositions, such as the *ACE1* DD phenotype or its combination with other genetic traits, and that SARS-CoV-2 infection is the final trigger for the fatal onset of COVID-19, is acceptable, it makes sense that similar situations can occur with many other illnesses.

Most medical disorders are caused by a combination of multiple genetic and/or environmental factors. This is natural given the complex structure of the human body and its integrity, which is composed of many organs and systems, such as the circulatory, nervous, and immune systems. Multifactorial disease is a disorder caused by multiple factors and mechanisms, including genes that act in concert with environmental and lifestyle factors. Therefore, a process of complicated interplays must occur in an individual for comorbidities or chronic diseases to develop. If the entire immune system is atrophied, including the T-cell repertoire, due to immune senescence, infectious diseases and malignant tumors will develop. This suggests that early immune aging for any reason is associated with the cumulative effects of genetic factors, lifelong inflammatory events including infections, and metabolic disorders. However, lifestyle changes may reduce immune aging, the cumulative effects, and the risk of developing disease. Therefore, to prevent exacerbations from comorbidities, it is necessary to find ways to avoid the risk factors associated with chronic inflammation by resolving inflammation at the right time and to balance proinflammatory and anti-inflammatory reactions. To achieve this goal, extensive research is required.

## Abbreviations

ARDS	acute respiratory distress syndrome
ADAM17	ADAM metallopeptidase domain 17
AAT	alpha-1 anti-trypsin
Ang I	angiotensin I
Ang II	angiotensin II
ACE1	angiotensin-converting enzyme 1
ACE2	angiotensin-converting enzyme 2
ABC	ATP-binding cassette
COPD	chronic obstructive pulmonary disease
CRP	C-reactive protein
CFTR	cystic fibrosis transmembrane conductance regulator
EGFR	epidermal growth factor receptor
E	envelopes
ERK	extracellular signal-regulated kinase
HDL	high-density lipoprotein
HLA	human leukocyte antigen
IL	interleukin
JAK	Janus kinase
STAT	signal transducer and activator of transcription
LGI	low-grade inflammation
MERS	Middle East respiratory syndrome
Nsps	non-structural proteins
NF-kB	nuclear factor kappa B
N	nucleocapsids
Orf	open reading frame
PRRs	pattern recognition receptors
ROS	reactive oxygen species
RAAS	renin–angiotensin–aldosterone system
RdRp	RNA-dependent RNA polymerase
SARS	severe acute respiratory syndrome
SARS-CoV-2	severe acute respiratory syndrome coronavirus 2
STAT	signal transducer and activator of
SHS	Suboptimal Health Status
TNF	tumor necrosis factor
TNFR	tumor necrosis factor receptor
